# Blockade of ubiquitin receptor Rpn13 in plasmacytoid dendritic cells triggers anti-myeloma immunity

**DOI:** 10.1038/s41408-019-0224-6

**Published:** 2019-08-12

**Authors:** Arghya Ray, Yan Song, Dharminder Chauhan, Kenneth C. Anderson

**Affiliations:** The LeBow Institute for Myeloma Therapeutics and Jerome Lipper Myeloma Center, Department of Medical Oncology, Dana Farber Cancer Institute, Harvard Medical School, Boston, MA USA

**Keywords:** Myeloma, Preclinical research

Dear Editor,

Proteasome inhibitors (PIs) represent a major advance in the treatment of relapsed/refractory and newly diagnosed multiple myeloma (MM)^1–3^; however, PI therapy can be associated with adverse effects and the emergence of drug resistance^2–4^. The potential tumor-intrinsic mechanism(s) underlying PI resistance include point mutations in 20S β-5 chymotrypsin-like (PSMB5) proteasome activity causing inefficient binding of PIs^5^; incomplete and/or transient inhibition of proteasomal activity^5^; induction of compensatory feedback pathways, such as via Nrf1, triggering transcriptional upregulation of proteasome catalytic subunits/proteasomal activities in response to PIs^6^; suppression of 19S subunits (e.g., PSMD5 via DNA methylation)^7^; activation of aggresome/autophagy signaling upon proteasome inhibition; as well as upregulation of MARKS, XBP1, and heat-shock proteins^7^. We and others have recently reported that targeting ubiquitin receptor (UbR) Rpn13 upstream of the 20S proteasome in the ubiquitin proteasome pathway can trigger cytotoxicity and overcome tumor-intrinsic PI-resistance mechanisms in MM^8,9^.

Importantly, tumor-extrinsic factors, such as sequelae of interactions of tumor cells with host-MM bone marrow (BM) accessory cells and immune effector cells, also promote drug resistance, immune suppression, and MM progression. For example, our recent studies show that interactions of dysfunctional plasmacytoid dendritic cells (pDCs) with MM cells and T/NK effector cells in the BM microenvironment, both confer immune suppression via immune checkpoints, as well as induce MM cell proliferation by triggering secretion of growth factors (e.g., IL-6, IGF-1, and TNF-α) and pro-survival signaling pathways (e.g., MAPK, PI3K/Akt, NF-κB, and IGF-1/IGF-1R). Importantly, our coculture models of patient pDCs, T cells, or NK cells with autologous MM cells^10,11^ can both delineate mechanisms of immunosuppression and validate targeted novel therapies to restore anti-MM immunity. For example, using these models, we previously showed that maturation/activation of MM patient pDCs via TL7/9 agonist or anti-PD-L1 Abs can restore their ability to induce T-cell proliferation^10–14^.

As noted above, we have shown that targeting ubiquitin receptor Rpn13 can overcome the intrinsic mechanisms of PI resistance, and here utilize our models of the BM milieu to examine immune sequelae of Rpn13 inhibition. We first assessed whether Rpn13 inhibition affects maturation of pDCs. MM patient pDCs were treated with nontoxic concentrations of a biochemical inhibitor of Rpn13 RA190 (0.05 µM)^8,9^, and examined for alterations in activation/maturation markers on MM pDCs. RA190 triggers significant upregulation of CD80, CD83, and CD86 on MM pDCs (Fig. 1a). In contrast to RA190, bortezomib-treated pDCs showed no significant upregulation of these markers (Fig. 1a). We confirmed our findings using RNA interference strategy: MM patient pDCs were transfected with Rpn13-siRNA, followed by flow cytometric analysis. As shown in Fig. 1b–d, genetic knockdown of Rpn13 significantly increased CD80 (*p* = 0.013), CD83 (*p* = 0.0068), and CD86 (*p* = 0.0014) expression in pDCs. Together, these findings suggest that RA190 triggers activation of MM patient pDCs. Although both RA190 and bortezomib block proteasome-mediated protein degradation, only RA190 triggers pDC activation.

**Fig. 1:**
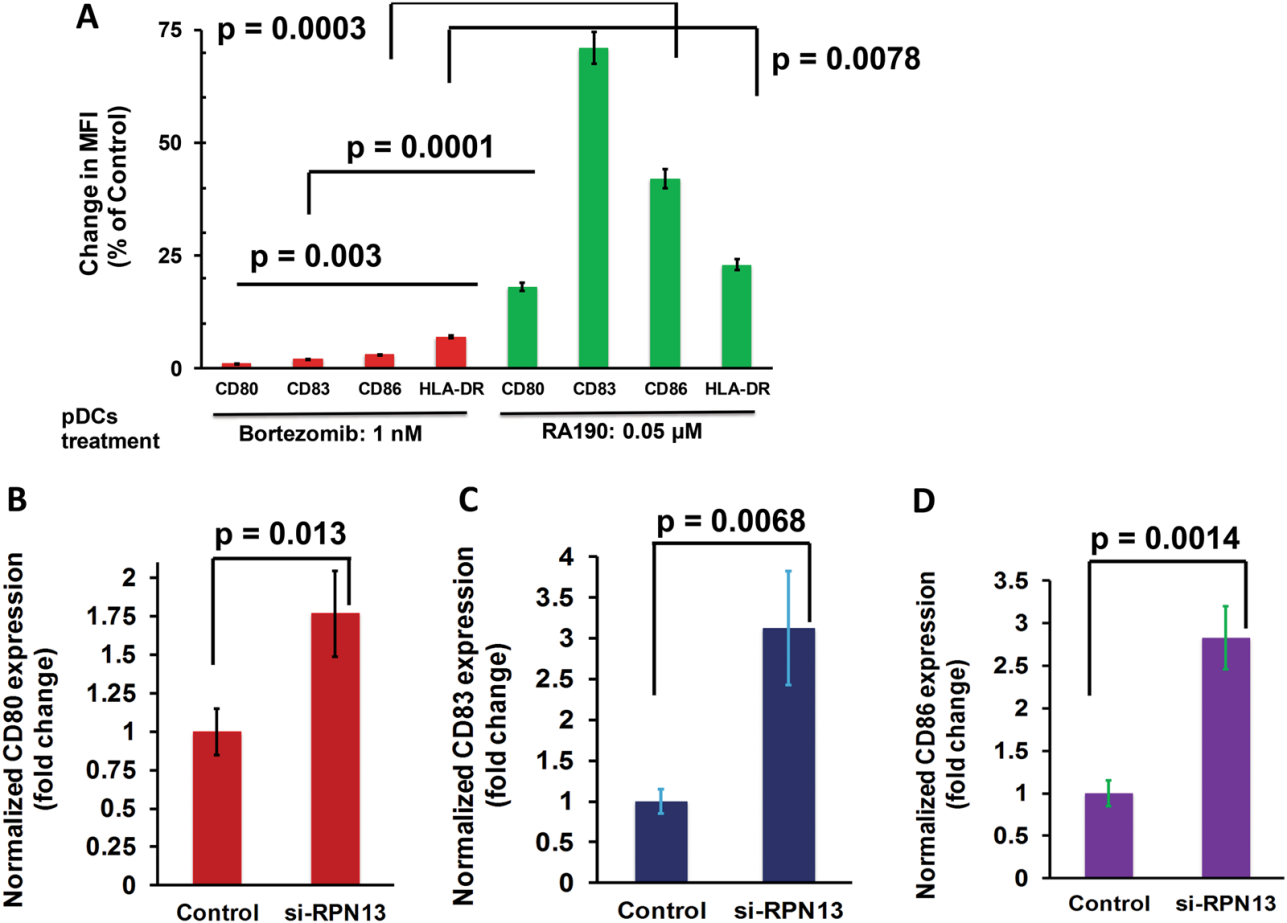
Biochemical or genetic blockade of UbR Rpn13 activates MM patient pDCs. **a** MM BM-pDCs were treated with RA190 (0.05 µM; green bars) or bortezomib (1 nM; red bars) for 24 h, followed by multicolor staining using fluorophore-conjugated Abs against the pDC activation/maturation markers CD80, CD83, and CD86. Bar graph shows the percentage change in median fluorescence intensities (MFI) for indicated markers in untreated- vs. bortezomib- or RA190-treated pDCs (Data obtained from BM samples from five different MM patients; mean ± SD; CD80: *p* = 0.003; CD83: *p* = 0.0001; CD86: *p* = 0.0003). **b**–**d** MM patient pDCs were transfected with Rpn13-siRNA using TransIT-X2 transfection reagent following the supplier’s protocol (Mirus Bio, USA). The transfected cells were cultured in complete DCP medium for 2 days, followed by multicolor staining with fluorophore-conjugated antibodies against CD80, CD83, CD86, and flow cytometry analysis. Bar graph: Quantification of MFI for each marker and data is shown as fold change between scrambled-siRNA- vs. Rpn13-siRNA-transfected pDCs. Analysis was performed using three different MM patient samples (mean ± SD; CD80: *p* = 0.013; CD83: *p* = 0.0068; CD86: *p* = 0.0014). Student’s *t*-test was utilized to derive statistical significance. The minimal level of significance was *p* < 0.05 (Graph Pad PRISM version 6, La Jolla, California, USA)

Using our coculture models, we next directly assessed whether Rpn13 blockade-induced pDC activation restores their ability to induce MM-specific cytotoxic T lymphocyte (CTLs). Freshly isolated MM patient BM CD8^**+**^ T cells were co-cultured with autologous pDCs (*n* = 8) at 1:10 (pDC:T cell) ratio in the presence or absence of RA190 (100 nM) for 3 days. After washing to remove RA190, these cells were cultured for 24 h with autologous MM cells pre-stained with CellTracker Violet (T/MM; 10:1 ratio), followed by 7-AAD staining and quantification of CTLs-mediated MM cell lysis using flow cytometry. As shown in Fig. 2a (scatter plot and bar graph), RA190 triggers significant MM-specific CD8^+^ CTL activity (*p* = 0.0015), evidenced by decreased viable patient MM cells. Among eight patient samples analyzed, two patients had newly diagnosed untreated MM, and relapsed MM was resistant to bortezomib, dexamethasone, and lenalidomide therapies in six patients.

**Fig. 2:**
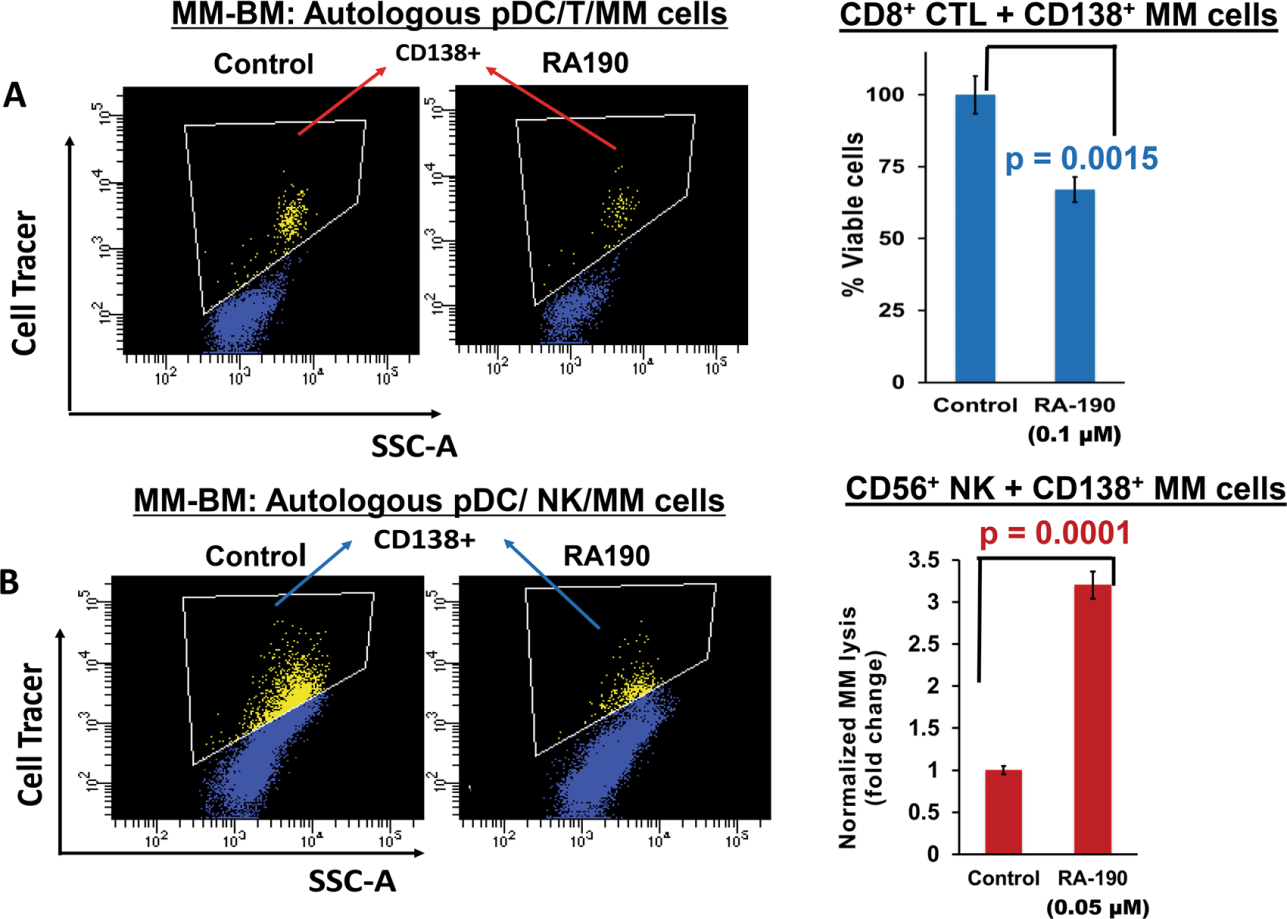
Rpn13 blockade triggers pDC-induced MM-specific CD8^+^CTLs and NK cell-mediated lysis of MM cells. **a** MM patient BM T cells were co-cultured with autologous pDCs (*n* = 8) at 1:10 (pDC:T cell) ratio in the presence or absence of RA190 (100 nM) for 3 days. After washing to remove RA190, cells were cultured with autologous MM cells pre-stained with CellTracker Violet (T/MM; 10:1 ratio) for 24 h, followed by 7-AAD staining and quantification of CTLs-mediated MM cell lysis by FACS. *Left panel*: Representative FACS scatter plot showing the decrease in number of viable CellTracker-positive MM cells. *Right panel***:** Bar graph shows quantification of CD8^**+**^ CTLs-mediated MM cell lysis, reflected in CD138^**+**^ MM cell viability. Data were obtained from eight different MM patient BM samples (mean ± SD; *p* < 0.05). **b** MM patient BM NK cells were co-cultured with autologous pDCs at 1:10 (pDC:NK cell) ratio in the presence or absence of RA190 (50 nM) for 3 days. After washing to remove RA190, cells were cultured with autologous MM cells pre-stained with CellTrace violet (10:1 NK cell:MM cell ratio) for 24 h, followed by 7-AAD staining and quantification of MM cell lysis by FACS. *Left Panel*: Representative FACS scatter plot showing a decrease in number of viable CellTrace Violet-positive MM cells. *Right Panel:* Bar graph shows quantification of NK-mediated MM cell lysis. The fold change was normalized with control, and MM cell lysis in RA190-treated vs. -untreated is presented. Data were obtained from four different MM patient BM samples (mean ± SD; *p* < 0.05). Student’s *t* test was utilized to derive statistical significance, and the minimal level of significance was *p* < 0.05 (Graph Pad PRISM version 6, La Jolla, California, USA)

In addition to pDCs, NK cells in MM also show immune dysregulation^11–14^. We therefore next examined whether Rpn13 inhibition alters anti-MM activity of NK cells using our autologous pDCs-NK cells-MM cells co-culture models. Freshly purified NK cells from MM patient BM were co-cultured with autologous pDCs in the presence of RA190 or DMSO control for 3 days; these cells were then washed to remove drug and cultured for 24 h with autologous MM cells. Importantly, RA190 also significantly increased NK cell cytolytic activity against MM cells (Fig. 2b, scatter plot and bar graph). Together, our results show that inhibition of Rpn13 activates pDCs and restores both pDC-induced T cell- and NK cell- mediated cytolytic activity against MM cells.

We next examined the mechanism(s) whereby RA190-activated pDCs stimulate MM-specific CTLs and NK cells. Earlier reports showed that lentiviral-mediated expression of calnexin (CNX), a molecular chaperone protein of endoplasmic reticulum (ER), in MM patient DCs triggers anti-MM CTL activity^15^. Another study showed that DCs with high calnexin expression stimulated expansion of high-avidity CTLs with increased central memory phenotype^15^. In this context, our previous study showed that RA190 induces ER stress response signaling including CNX in MM cells^8^. Based on these reports, we next examined whether Rpn13 blockade via RA190 alters calnexin levels. Our studies showed that treatment of MM patient pDCs with RA190 increases calnexin expression (Supplementary Fig. 1A). Similar results were observed using plasmacytoid dendritic cell line Cal-1 (Supplementary Fig. 1B). Furthermore, nontoxic concentrations of RA190 also induced calnexin expression in MM cells (Supplementary Fig. 1C). Prior studies have shown that CNX upregulates co-stimulatory molecules, cytokines/chemokines, adhesion molecules, and immune signaling pathways^15^. Rpn13 inhibition-triggered CNX expression may alter immune-regulatory signaling, thereby restoring the ability of pDCs to induce proliferation of MM-specific CTLs. Our ongoing studies are elucidating the RA190-induced immune signaling cascades in the MM BM milieu.

In summary, we here show that pharmacological or genetic inhibition of ubiquitin receptor Rpn13 activates MM patient pDCs as well as triggers MM-specific CTLs and NK-cell-mediated cytotoxicity against autologous MM cells. Our study highlights the therapeutic potential of targeting ubiquitin receptor Rpn13 to abrogate immune suppression and restore both innate (pDCs) and adaptive immune responses (T and NK cells). Our prior findings showed that inhibition of UbR Rpn13 overcomes intrinsic PI-resistance^8^ in MM cells. Rpn13 blockade therefore represents a novel therapeutic approach to overcome both PI resistance and immune suppression in MM.

## Supplementary information

Supplemental Data

Supplementary Figure S1
