# Supplementary material for: Blockade of ubiquitin receptor Rpn13 in plasmacytoid dendritic cells triggers anti-myeloma immunity
Source: Blood Cancer J. 2019 Aug 12;9(8):64. doi: 10.1038/s41408-019-0224-6 (PMC6690908; doi:10.1038/s41408-019-0224-6)
Supplement: Supplementary file 1 — Supplemental Data [file 41408_2019_224_MOESM1_ESM.doc]

## Supplemental Data

**Blockade of Ubiquitin Receptor Rpn13 in Plasmacytoid Dendritic Cells Triggers Anti-Myeloma Immunity**

Short Title: Inhibition of Rpn13 as immunotherapy in multiple myeloma

Arghya Ray, PhD, Yan Song, PhD, Dharminder Chauhan*¶, PhD and Kenneth C Anderson*¶, MD.

The LeBow Institute for Myeloma Therapeutics and Jerome Lipper Myeloma Center, Department of Medical Oncology, Dana Farber Cancer Institute, Harvard Medical School, Boston, MA

***¶****Joint Senior authors*

**Grant Support**: This investigation was supported by National Institutes of Health Specialized Programs of Research Excellence (SPORE) grant P50100707, RO1 CA207237 (DC and KCA), and RO1 CA050947 (KCA). KCA is an American Cancer Society Clinical Research Professor.

**Conflicts of Interest disclosure**. KCA is on Advisory Boards of Celgene, Millenium-Takeda, Gilead, Janssen, Sanofi-Aventis, and Bristol Myers Squibb; and is a Scientific Founder of Oncopep and C4 Therapeutics. DC is consultant to Stemline Therapeutics, Inc., and Equity owner in C4 Therapeutics. The remaining authors declare no conflict of interest. Other authors have no competing financial interests.

*** Correspondence** [Dharminder_Chauhan@dfci.harvard.edu](mailto:Dharminder_Chauhan@dfci.harvard.edu); [Kenneth_Anderson@dfci.harvard.edu](mailto:Kenneth_Anderson@dfci.harvard.edu) Dana-Farber Cancer Institute, M561, 450 Brookline Ave, Boston, MA; #DC: Ph: 617-632-4563; KCA: Ph: 617-632-2144; Fax#: 617.632.2140

**Keywords:** Myeloma, Immunotherapy, Plasmacytoid Dendritic Cells, Ubiquitin Receptor Rpn13/ADRM1

**Word count: 1208; Number of Figures: 2; Supplementary Figure: 1; Number of References 15**

**Scientific Category: Multiple Myeloma**

**Blockade of Ubiquitin Receptor Rpn13 in Plasmacytoid Dendritic Cells Triggers Anti-Myeloma Immunity**

**Correspondence**

**Supplementary Figure Legend**

Supplementary Figure 1 **Rpn13 inhibition by RA190 triggers Calnexin (CNX) expression (A)** MM patient pDCs cells were treated with RA190 (0.05 µM) or DMSO control for 16h. After washing to remove drug, cells were stained with Calnexin (CNX) or isotype control Abs conjugated with AlexaFluor-647, and subjected to flow cytometry analysis. Bar graph shows quantification (MFI) of CNX expression after normalization with isotype control Ab. The fold change in CNX levels in RA190-treated versus -untreated is presented (mean ± SD; p < 0.05; n = 3). **(B)** Blastic plasmacytoid dendritic cell neoplasm (BPDCN) Cal-1 cells were treated with RA190 (0.05 µM) or DMSO control for 16h. After washing to remove drug, cells were stained with Calnexin (CNX) or isotype control Abs conjugated with AlexaFluor-647, and subjected to flow cytometry analysis. Bar graph shows quantification (MFI) of CNX expression after normalization with isotype control Ab. The fold change in CNX levels in the RA190-treated versus -untreated is presented (mean ± SD; p < 0.05; n = 3). **(C)** MM patient (CD138**^+^**) cells were treated with of RA190 (0.05 µM) or DMSO control for 16h. After washing to remove drug, cells were stained with Calnexin (CNX) or isotype control Abs conjugated with AlexaFluor-647, and subjected to flow cytometry analysis. Bar graph shows quantification (MFI) of CNX expression after normalization with isotype control Ab. The fold change in CNX levels in the RA190-treated versus -untreated is presented (mean ± SD; p < 0.05; n = 3).

**Supplementary File**

**Materials and Methods**

**Purification of MM patient Bone marrow plasmacytoid dendritic cells (pDCs), T cell, NK cells, and MM cells** All studies with MM patient samples were done following IRB-approved protocols (Dana-Farber Cancer Institute/Brigham and Women’s Hospital, Boston, USA). Informed prior consent was obtained from all patient in accordance with Helsinki protocol, and patient samples were de-identified prior to their use in assays. pDCs were isolated from bone marrow (BM) using CD304 (BDCA-4/Neuropilin-1) microbeads kit (Miltenyi Biotec). The purity of pDCs (CD3-, CD14-, CD19-, CD20-, CD56-, CD11c-, MHC-II/CD123/BDCA-2+) was confirmed by flow^10,11^. The data obtained from flow experiments were analyzed using FACS Diva (BD Biosciences) and FlowJO (Tree Star Inc, USA) softwares. MM patient cells were purified (>95% purity) by positive selection using CD138 Microbeads kit (Miltenyi Biotec Inc). T and NK cells were purified using negative selection immunomagnetic separation techniques (Ab-Microbeads kits; Miltenyi Biotec). The purity was confirmed by flow, as previously described^10,11^. CD3-PE/FITC/APC; CD4-FITC/PE or APC-Cy7; CD8-APC/FITC, CD56-PE; CD123-PE/PE-Cy5/FITC; and CD138-FITC/PE/APC were obtained from BD Biosciences (San Jose, CA, USA). BDCA-2-FITC, and CD11c-APC were obtained from Miltenyi Biotec (Auburn, CA); CD304; CD107a; and CD303--BV421 Abs were purchased from Biolegend. Calnexin Ab was from Novus Biochemicals.

**Statistics** Student’s *t* test was applied to derive statistical significance. All statistical analyses were calculated with GraphPad Prism software.
